# Child Opportunity Index Predicts Outcomes in Pediatric Spine Trauma: A Novel Application of Social Determinants of Health

**DOI:** 10.3390/children12030380

**Published:** 2025-03-19

**Authors:** Gabriel Urreola, Omar Ortuno, Michael Juma, Jose Castillo

**Affiliations:** Department of Neurosurgery, University of California, Davis, Sacramento, CA 95618, USA; oaortuno@ucdavis.edu (O.O.); mojuma@ucdavis.edu (M.J.); jcastillo@ucdavis.edu (J.C.)

**Keywords:** pediatric, spine trauma, social disadvantage index, child opportunity index, epidemiology, socioeconomic, disparities, insurance

## Abstract

**Objectives:** Social factors play a crucial role in health outcomes for pediatric patients, yet in the neurosurgery pediatric literature, these factors are rarely reported. To develop a deeper understanding of pediatric spine trauma outcomes, we investigate demographic and social factors measured by the Child Opportunity Index (COI) and Social Deprivation Index (SDI). We hypothesize that social factors predict clinical presentation, injury severity, and clinical outcomes. **Methods:** We conducted a retrospective cohort study of pediatric patients treated for spinal trauma at a Level 1 trauma center in Sacramento, California. We collected patient clinical data such as mechanisms of injury (MOIs), length of stay (LOS), treatment type, hospital disposition, polytrauma incidence, and follow-up attendance. Each patient’s social environment was characterized using COI and SDI metrics. Statistical comparisons were performed to assess associations between social factors and clinical outcomes. **Results:** Patients with worse childhood opportunity (lower COI and higher SDI) were more likely to be insured through Medi-Cal, identify as Hispanic, and experience violent MOI. Female patients were more likely to sustain polytrauma and had a higher likelihood of requiring surgical intervention. Additionally, patients from underserved communities demonstrated longer hospital stays and poorer follow-up adherence, with COI and SDI scores correlating with these disparities. **Conclusion**: Social disparities are associated with worse outcomes in pediatric spine trauma. We found COI and SDI to be valuable clinical metrics, motivating further research to be carried out at the state and national levels. These findings highlight health disparities in pediatric spine trauma.

## 1. Introduction

Social factors play a crucial role in health outcomes, with pervasive associations with mechanisms of injury, injury severity and subsequent management [1,2]. Despite the clinical significance of social factors, very few publications report how these factors impact pediatric patients undergoing neurosurgical care [3,4]. Understanding health disparities in neurosurgical care is essential because pediatric patients make up 30% of all neurosurgery and close to 10% of all United States pediatric surgery [2]. Regarding spine trauma, injury mechanisms are well understood, with falls and pedestrian accidents being more common in young children and sport injuries and motor vehicle collisions occurring more frequently in older children [4].

Despite understanding injury mechanisms, the social and demographic factors and the corresponding clinical implications are much less defined in the literature. Epidemiological data on pediatric spinal trauma is sparse, despite injury prevention affording a large opportunity in healthy person years and economic costs saved [5,6]. To our knowledge, there have not been institution-specific data published on social factors and pediatric spine trauma. Moreover, there is a gap in knowledge regarding how social factors such as sex, race, and SES proxies (SDI and COI) interplay with clinical outcomes like length of stay, ICU admissions, and follow-up completion. The Social Deprivation Index (SDI) is a composite measure of social deprivation based on demographics that is used to quantify socioeconomic variation in health outcomes [2]. Similarly, the Child Opportunity Index (COI) is a composite index of a children’s neighborhood opportunity that comprises three domains—education, health and environment, and social and economic [7].

The primary goal of this study is to identify any racial, sex- or insurance-related, or other socioeconomic inequity in the healthcare of pediatric spine trauma. We hypothesized that differences exist in social economic factors, as measured by COI and SDI, when it comes to pediatric spine trauma patients. We postulate that these social determinants can potentially serve as additional predictors of clinical outcomes in pediatric trauma patients and can underscore the need to include these metrics as an outcome measure for future studies at the state and national levels.

## 2. Materials and Methods

An IRB-approved retrospective chart review of a prospectively maintained database was performed looking at pediatric patients (<18 years of age) who sustained traumatic spine injury during the pre-COVID-19 era of 2015–2019. Patient demographic data including age, gender, zip code, sex, and insurance were extracted from the database. Additional clinical data that detailed the nature of trauma sustained by each patient such as the mechanism of injury, spinal cord injury, associated injuries, ICU status, length of stay, post-hospitalization consults/follow-up, and disposition were also collected. Patients were categorized further as to whether they were transferred from another institution or directly admitted to our center. Socioeconomic data based on the Child Opportunity Index (COI) and Social Deprivation Index (SDI) were determined for each patient entered in the database [2,7].

COI data were extracted from the Childhood Opportunity 2.0 database constructed by the Institute for Child, Youth, and Family Policy, the Heller School for Social Policy and Management [5]. The COI measures the level of opportunity that neighborhoods provide children in the United States. There are 29 indicators that comprise the COI 2.0 scores and are grouped into one overall score as well as three domains which include education (E), health and environment (HE), and social and economic (SEC). Similarly, SDI data were used as a composite measurement of child deprivation based on 7 key domains: percent living in poverty, percent with less than 12 years of education, percent single-parent households, the percentage living in rented housing units, the percentage living in an overcrowded housing unit, percent of households without a car, and percentage nonemployee adults under 65 years of age [2]. SDI data were extracted from the Robert Graham 2019 dataset [8] utilizing the zip code tabulation area (ZCTA) to determine SDI scores for each patient [4]. COI and SDI scores were determined for each patient.

By referencing the SDI and COI against treatment type, LOS, disposition, and follow-up attendance, we were able to determine whether differences existed amongst low deprivation indices or in higher-deprivation areas. Similarly, the differences in treatment type based upon insurance type, i.e., private versus state-funded insurance (Medi-Cal or the California State Crippled Children’s Services), were examined to determine any skewing towards different income levels.

Clinical and demographic data were analyzed by performing chi-square tests for categorical data, independent *t*-tests, one-way analysis of variance (ANOVA), and linear regression for continuous data using JASP statistical analysis software (Version 0.19.3.0) [9]. Significance was set at 0.05.

## 3. Results

**Figure 1:**
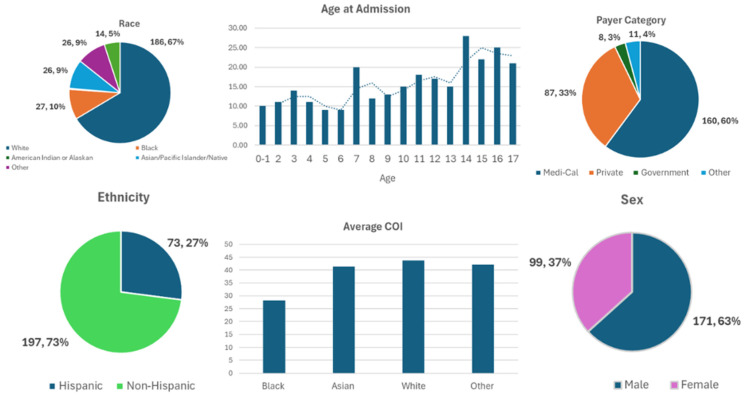
Patient demographics: race, age at admission, payer category, ethnicity, average COI, and sex. The dotted line demonstrates a trend-line of average age at admission.

Table 1 and Figure 1 demonstrate the general demographic and social factors of our patient population. Our database of pediatric spine trauma from 2015 to 2019 had a total of 270 patients with an average age of 10.4 ± 4.8 years and average hospital LOS of 4.7 ± 6.6 days (Table 1). For patients admitted to the ICU, average LOS was 3.22 ± 4.19 days. Most patients were non-Hispanic (73%), white (68.9%), and males (63.3%). Racial categories identified included 68.9% white, 10% Black, 0.4% American Indian, and 9.6% Asian/Pacific Islander/Native Hawaiian, and the rest were either unreported, unknown or “other’” (Table 1). Most patients had Medi-Cal Insurance (59.3%), with a lesser but significant number of patients having private coverage (32.2%).

Table 2 provides insight into the social determinants of health of patients included in our study. The average COI of pediatric patients with spine trauma for education, health and environment, and socioeconomics was 38.29 ± 30.51, 55.83 ± 22.42, and 40.82 ± 26.79, respectively. The overall COI was found to be 42.10 ± 60.33, with the average SDI being 60.33 ± 28.34. The average state of California COI and SDI scores acted as references and were 51.5 and 52.9, respectively. Our data demonstrate that, on average, pediatric patients who present with spine trauma have worse social environments relative to the state average. Table 3 demonstrates the relationship between COI and social factors and spinal trauma characteristics. As determined by the COI and SDI scores, Hispanic children in addition to children with Medi-Cal Insurance had statistically significant deficiencies in childhood opportunities (*t*-test, *p*-value < 0.001) and increased social disadvantage (*t*-test, *p*-value < 0.001). Additionally, as the COI decreased patients were more likely to have sports or violent injuries (i.e., gunshots, stabbings, assault) as the MOI. In contrast, as COI increased, patients were more likely to have falls or motor vehicle accidents as their MOI (ANOVA, *p*-value 0.047; falls: 47.4 ± 29.4; MVA: 39.7 ± 27.36 vs. sport: 30 ± 22.4; violent: 22 ± 16.14, *p*-value 0.047). Of note, regardless of MOI, the average COI/SDI was below the average state of California scores. Patients with lower COI were more likely to be lost to follow-up at 12 months (*t*-test, COI 40.11 ± 27.27 lost to follow up vs. COI 46.3 ± 28.15 with follow-up, *p*-value 0.04).

Table 4 explores how sex affects social factors and trauma characteristics. Of the patients admitted, males represented 63.3%, with females being 36.7%. Female patients were more likely to sustain polytraumatic injuries (chi-square, 72% females vs. 55% males, *p*-value 0.007) and undergo spine surgery despite similar mechanisms of injury. Although more males were admitted with traumatic spinal injuries in our sample population, there were no significant differences when compared to females regarding length of stay, age, ethnicity, race and insurance status. No differences were appreciated between demographic variables and disposition.

Table 5 demonstrates the interplay between insurance status and social factors and trauma characteristics. A variety of payer categories were identified in our sample population including Medi-Cal and various private insurance providers (Table 5). Hispanic patients were more likely to have Medi-Cal Insurance when compared to their non-Hispanic counterparts (chi-square, 70% of Hispanics vs. 46% of non-Hispanics, *p*-value 0.05). Pediatric patients with Medi-Cal also tended to be younger (average age of 9.86 years) than those with private coverage (average age of 11.45) (*t*-test, *p*-value 0.01). Moreover, Medi-Cal patients were found to have a lower COI (*t*-test, COI 34.12 ± 27.6, *p*-value < 0.001) and a higher SDI (*t*-test, SDI 69.8 ± 28.3, *p*-value < 0.001) when compared to patients with private insurance (COI 54.21 ± 28.9, SDI 46.73 ± 28.3).

Lastly, Table 6 explores the relationship between ethnicity and trauma outcomes. Notably, Hispanic patients were more likely to be sent directly from the scene to our medical center than their non-Hispanic counterparts despite having similar mechanisms of injury (chi-square, 61% of Hispanics vs. 55% of non-Hispanics, *p*-value 0.002) (Table 6). Hispanic patients were also more likely to have longer ICU stays (*t*-test, 4.29 ± 5.46 days in Hispanics vs. 2.76 ± 3.46 days in non-Hispanics, *p*-value 0.048).

## 4. Discussion

**Figure 2:**
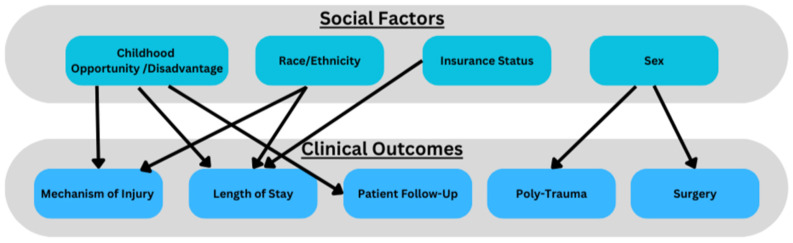
Interplay between social factors and clinical outcomes.

This study used a single-institution database to investigate the interplay between social factors and spinal trauma in pediatric patients. Lechtholz-Zay et al. demonstrate only one publication investigating how socioeconomic factors interplay with spinal injuries [1]. The study demonstrated, from a large public database, that mechanisms, patterns and injury severity had important associations with age, race and payor [11]. Our study demonstrates that ethnicity, sex, insurance status and SES (measured by COI/SDI) is associated with initial clinical presentation and subsequent clinical outcomes in pediatric patients with spinal trauma. We found COI and SDI to be effective metrics for social determinants of health and gauging clinical outcomes. For instance, patients from lower-deprivation areas (low COI, high SDI) were more likely to be Hispanic, have government-provided insurance (Medi-Cal), experience spinal trauma from violent mechanisms of injury, and have longer ICU length of stays. Our data align with national data that low socioeconomic status is associated with poorer trauma outcomes [4]. In contrast, patients from higher-SES backgrounds were more likely to sustain injuries from motor vehicle accidents or falls. These findings demonstrate associations with poor social factors and pediatric spinal trauma, where children from less fortunate backgrounds face a higher burden of severe injuries [12]. Given the significant interactions between social factors and clinical outcomes, we propose that pediatric spine trauma data/research can be conceptualized via frameworks of social determinants of health (Figure 2).

Additionally, Hispanic patients experienced longer ICU stays despite presenting with similar mechanisms of injury and injury severity as their non-Hispanic counterparts. Notably, among all social determinants examined, ethnicity emerged as the only factor significantly correlated with ICU length of stay. Neither polytrauma nor more violent or high-impact mechanisms of injury significantly influenced hospital or ICU length of stay durations. This aligns with previous research suggesting that ethnic and socioeconomic disparities contribute to differences in trauma outcomes [3]. However, insurance type (Medi-Cal vs. private) was not independently associated with differences in LOS or disposition, suggesting that other social determinants, beyond insurance status alone, play a role in shaping patient outcomes.

Our study also identified gender-based differences in pediatric spinal trauma outcomes, with female patients more likely to experience polytrauma and require surgical intervention. While prior research suggests that males are generally at higher risk for spinal trauma due to greater engagement in risk-taking behaviors, our findings indicate that female patients who do sustain spinal trauma may have more severe injuries, potentially due to unclear social or physiological factors [13,14]. Bilston et al. demonstrated that all spinal injuries between sexes were approximately equal, while our data suggest that there may be other underlying mechanisms impacting clinical outcomes [14]. Our data call to question the interplay between sex, SES and spine trauma and the need for further research (Figure 2).

Patients from lower-deprivation areas had lower follow-up adherence at 12 months. These findings suggest that structural barriers, such as financial constraints, transportation issues, or healthcare access disparities, may hinder long-term recovery for these patients [15]. Previous research demonstrates that language and cultural barriers often present challenges in patient adherence and follow-up [16]. Of note, our data do not show significant difference between follow-up rates and race or ethnicity, but we do see differences when factoring in social deprivation metrics. Our findings are congruent with other published reports where race and low SES are associated with poor outcomes in trauma patients [10,17,18,19]. Identifying and addressing these barriers could improve post-discharge outcomes in vulnerable pediatric populations.

Future research should establish comprehensive, multicenter databases that prospectively collect both socioeconomic metrics and clinical outcomes in pediatric spine trauma patients. Specifically, objective metrics such as Injury Severity Score (ISS) would be effective in further analyzing social factors and how that may impact injury severity—a limitation of our current study. These longitudinal studies should track patients from injury through rehabilitation and long-term follow-up.

We propose several potential interventions to address the disparities identified in our study. We suggest implementing standardized screening protocols for social vulnerability in pediatric trauma centers, with automatic triggers for additional support resources when needed. Further research needs to evaluate why the long-term 12-month follow-up that our study observed was worse in socioeconomically vulnerable populations. An example of this is transportation services and at-home nurse visits or telehealth. Partnerships with community outreach programs could target high-risk individuals, providing preventative education on activities and avenues that increase risk of spinal trauma. We found these avenues to be sports and violence.

From a policy perspective, our findings support the need for healthcare reimbursement models that account for the additional resources required to achieve equitable outcomes for patients from disadvantaged backgrounds. This could include enhanced reimbursement for hospitals serving predominantly low-COI/high-SDI populations and incentives for maintaining robust follow-up programs for vulnerable patients. Our findings suggest that trauma centers should consider socioeconomic factors when developing discharge planning and follow-up protocols, with extra attention to populations identified as high-risk in our analysis. These groups include Hispanic/Black patients, socioeconomically disadvantaged patients, patients with public insurance and those who came in due to violent mechanisms of injury.

### Limitations

While our study provides valuable insights, several limitations should be considered. As a single-center study conducted at a Level 1 trauma center, the findings may not be fully generalizable to broader populations, necessitating validation through multi-center or statewide databases. Although our findings regarding sex differences in pediatric spine trauma outcomes motivate further research, we acknowledge our small sample size and the need for similar studies at a larger scale. A limitation of our study lies in our ethnicity and racial classification methodology. Our primary ethnic comparison was restricted to Hispanic versus non-Hispanic categories, which fails to capture the rich diversity and heterogeneity within these broad populations. Similarly, our racial categorization (white, Black, Native American, and Asian) lacks the granularity needed to identify important intra-group variations that may significantly influence clinical outcomes. Future research would benefit from prospective study designs with more comprehensive and nuanced race/ethnicity classifications, ideally utilizing larger multi-institutional datasets to overcome these limitations.

The retrospective nature of our study also introduces the possibility of unmeasured confounding variables that could influence outcomes; future prospective studies may provide a more precise understanding of the causal relationships between social determinants of health and spinal trauma. Moreover, future studies would benefit from multivariate analysis of specific sub-variables such as parent education, trauma center accessibility and hospital resources. Despite these limitations, our findings align with prior research emphasizing SES as a key determinant of pediatric trauma outcomes and support the use of COI and SDI as effective metrics for assessing social determinants of health in spine trauma research. 

## 5. Conclusions

Our data demonstrate that the socioeconomic profile of patients who experience pediatric spine trauma varies significantly. We found that Hispanic patients were more likely to come from socioeconomic disadvantage (low COI) and have longer lengths of stay. Female patients were more likely to sustain polytrauma and undergo surgery. Patients with a lower COI had worse 12-month follow-up adherence and were more likely to sustain injury from violent mechanisms. Lastly, we found that pediatric patients who experience spine trauma have lower COI and SDI metrics compared to the state average. We propose that many hospitalizations for pediatric spine trauma may act as opportunities to address health disparities or act as models for prevention.

## Figures and Tables

**Table 1:** Basic demographics.

Variable	Value
Total sample size	270
Age (years, SD)	10.4 ± 4.8
Gender	
Female	99 (36.7%)
Male	171 (63.3%)
Ethnicity	
Hispanic	73 (27.0%)
Non-Hispanic	197 (73.0%)
Race	
White	186 (68.9%)
Black	27 (10.0%)
American Indian or Alaska Native	1 (0.4%)
Asian/Pacific Islander/Native Hawaiian	26 (9.6%)
Other	16 (5.9%)
Not reported	11 (4.1%)
Unknown	3 (1.1%)
Payer Category	
Blank	4 (1.5%)
Medi-Cal	160 (59.3%)
Private Coverage	87 (32.2%)
Other Government	8 (3.0%)
Other pay	11 (4.1%)
Length of stay (days, SD)	4.7 ± 6.6

**Table 2:** General COI and SDI.

Variable	Value
COI	
Education Domain	38.29 ± 30.51
Health and Environment Domain	55.83 ± 22.42
Social and Economic Domain	40.82 ± 26.79
Overall COI	42.10 ± 60.33
Average California State COI [10]	51.5
SDI	60.33 ± 28.34
Average California State SDI [10]	52.9

**Table 3:** Comparison by COI.

Variable	COI	*p*-Value
Overall	42.10 ± 27.64	
Sex		
Male	42.88 ± 28.19	0.544
Female	40.75 ± 26.76	0.544
Payer Category		
Medical	34.12 ± 27.6	<0.001
Private	54.21 ± 28.9	<0.001
Ethnicity		
Hispanic	32.64 ± 27.57	<0.001
Non-Hispanic	45.71 ± 27.63	<0.001
Mechanism of Injury		
Fall	47.4 ± 29.4	0.047
MVA	39.7 ± 27.36	0.047
Sport	30 ± 22.4	0.047
Violent	22 ± 16.14	0.047
No—12-Month Follow-Up	40.11 ± 27.27	0.04
Yes—12-Month Follow-Up	46.3 ± 28.15	0.04

MVA—Motor Vehicle Accident.

**Table 4:** Comparison by gender.

Variable	Male	Female	*p*-Value
Sample Size	171	99	
Age	10.2 ± 4.9	10.6 ± 4.8	0.51
Payer Category			
Medical	98 (57.30%)	59 (60.0%)	0.23
Private Insurance	62 (36.26%)	27 (27.27%)	0.23
Length of Stay	4.45 ± 7.26	5.05 ± 5.3	0.47
Ethnicity			
Hispanic	44 (26.00%)	30 (30.3%)	0.42
Non-Hispanic	127 (74.30%)	69 (25.6%)	0.42
Race			
White	120 (70.20%)	66 (70.0%)	0.21
Black	17 (10%)	10 (10.1%)	0.21
Asian	11 (6.43%)	13 (13.1%)	0.21
Trauma Characteristics			
Vertebrae Injured	102 (60.0%)	73 (73%)	0.058
Polytrauma	94 (55.0%)	71 (72.0%)	0.007
Spine Surgery	18 (11.0%)	21 (21.21%)	0.016
No Spine Surgery	153 (90.0%)	78 (79.0%)	0.016
COI	42.88 ± 28.19	40.75 ± 26.76	0.544
SDI	59.33 ± 28.84	62.03 ± 27.50	0.454

**Table 5:** Comparison by payer category.

Variable	Medi-Cal	Private Coverage	*p*-Value
Sample Size	160	87	
Age	9.86 ± 4.8	11.45 ± 4.27	0.01
Gender			
Male	98 (61.30%)	59 (68.0%)	0.23
Female	62 (39.00%	27 (31%)	0.23
Length of Stay			
Hospital	4.86 ± 6.6	3.96 ± 5.5	0.30
ICU	2.75 ± 2.56	3.26 ± 5.065	0.44
Ethnicity			
Hispanic	52 (33.0%)	18 (21.0%)	0.05
Non-Hispanic	108 (68.0%)	69 (79.31%)	0.05
Race			
White	104 (65.0%)	65 (75.0%)	0.07
Black	19 (12.0%)	3 (4.0%)	0.07
Asian	15 (9.40%)	8 (9.2%)	0.07
COI	34.12 ± 27.6	54.21 ± 28.9	<0.001
SDI	69.8 ± 28.3	46.73 ± 28.3	<0.001

ICU—Intensive Care Unit.

**Table 6:** Comparison by ethnicity.

Variable	Hispanic	Non-Hispanic	*p*-Value
Sample Size	74	196	
Age	10.46 ± 4.82	10.31 ± 4.83	0.82
Payer Category			
Medical	52 (70.3%)	91 (46.43%)	0.05
Private Insurance	18 (24.32%)	59 (30.10%)	0.05
Gender			
Male	44 (60%)	67 (34.1%)	0.44
Female	30 (41.0%)	41 (21.0%)	0.44
Length of Stay			
Hospital	5.72 ± 6.61	4.272 ± 6.62	0.11
ICU	4.29 ± 5.46	2.76 ± 3.46	0.048
Transfer Status			
Direct from Scene	45 (61%)	108 (55.10%)	0.002
Transferred	25 (34.0%)	88 (45.0%)	0.002
COI	32.64 ± 27.57	45.71 ± 27.63	<0.001
SDI	70.32 ± 28.25	56.52 ± 28.33	<0.001

## Data Availability

The raw data supporting the conclusions of this article will be made available by the authors on request.

## Abbreviations

The following abbreviations are used in this manuscript:

SCI	spinal cord injury
SDI	Social Disadvantage Index
CDI	Child Opportunity Index
SES	socioeconomic status
